# Supplementary material for: BCAA (Branched-Chain Amino Acids) Inhibiting the Autophagy System via the Activation of mTORC1, Thereby Upregulating the Tumor Suppressor PDCD4 in Huh7 Hepatoma Cells
Source: Cells. 2025 Dec 11;14(24):1975. doi: 10.3390/cells14241975 (PMC12731450; doi:10.3390/cells14241975)
Supplement: Supplementary file 1 [file cells-14-01975-s001.zip › Supplementary figures.pdf]

Supplementary Materials

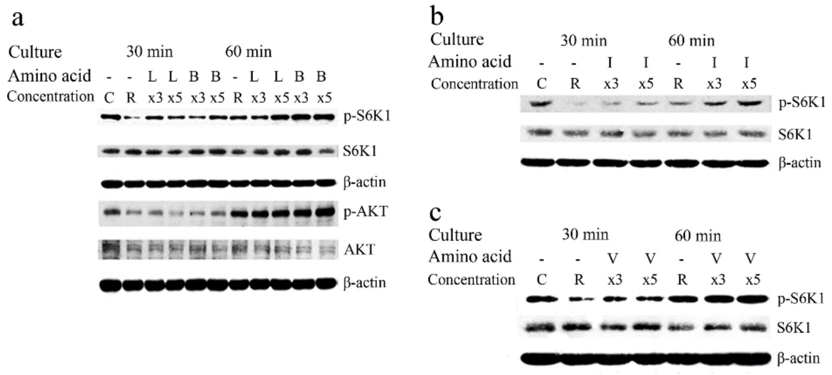

Figure S1. BCAA (B) and BCAA member amino acids leucine (L), isoleucine (I) and valine (V) upregulated the phosphorylation of p70S6K1 (S6K1) also in the RPMI (R) medium system. Huh7 cells were treated with BCAA or BCAA amino acids along with cells cultured in DMEM containing FBS (C) as described in the Materials and Methods, and analyzed by immunoblotting using the antibodies mentioned in the figures. (**a**) Leucine (L) and BCAA (B) upregulated the phosphorylation of p70S6K1 (pS6K1) with little phosphorylation of Akt. (**b**) Isoleucine (I) upregulated the phosphorylation of p70S6K1 (pS6K1). (**c**) Valine (V) upregulates the phosphorylation of p70S6K1 (pS6K1).

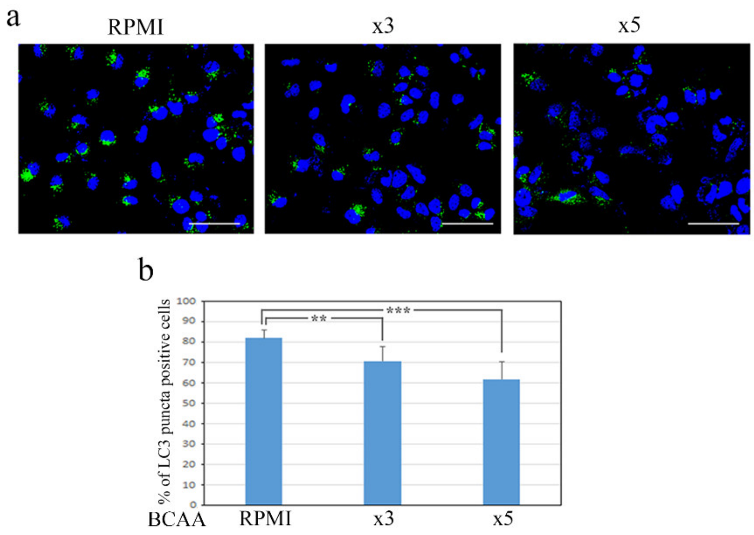

Figure S2. The formation of LC3 particles was inhibited by the BCAA treatment of Huh7 cells in the RPMI medium system. (**a**) Immunocytochemical staining of LC3 (green) with nuclei counterstained in blue. Huh7 cells were treated with RPMI (R), x3 BCAA, and x5 BCAA media and stained with rabbit anti-LC3 antibody, as described in the Materials and Methods section. Scale bar indicates 100µm. (**b**) The percentage of LC3-puncta-positive cells obtained from (**a**). 12 field containing 80-150 cells in each were photographed in different places, and LC3-particle-positive cells were counted. The results were then obtained as the mean ± SD of the number in the 12 fields. *t*-test: ** $p < 0.01$, *** $p < 0.001$.

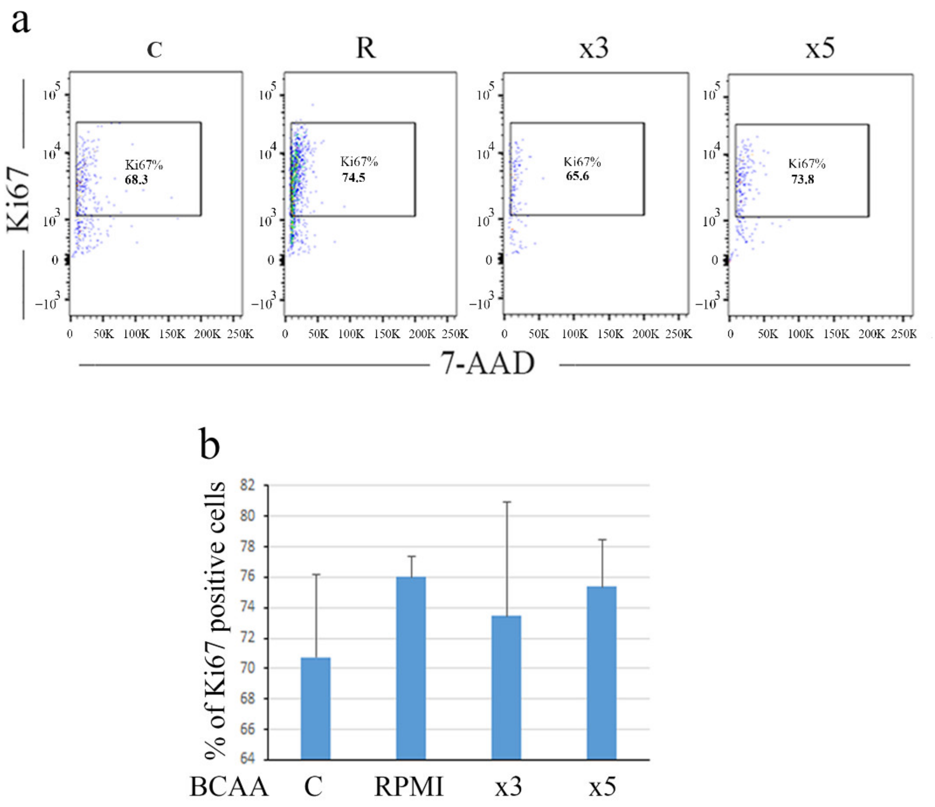

Figure S3: BCAA partially inhibited cell proliferation in Huh7 cells in RPMI system. (**a**) A FACS analysis of BCAA-treated Huh7 cells. After a 3-day culture, Huh7 cells were treated for 4 h with the RPMI system using different concentrations of BCAA [RPMI (R), x3, x5)] along with cells cultured in DMEM containing FBS (C). Cells were stained with anti-Ki67 antibody and 7-AAD as described in the Materials and Methods and analyzed by flow cytometry. After the removal of the dead and doublet cells through gating, singlet cells were channeled in a combination of Ki67 and 7-AAD staining. This experiment was repeated three times, and a representative gate of an experiment is shown. Each dot represents Ki67-positive single cell. (**b**) The diagram shows the average percentage of Ki67-positive cells in the three experiments.
